# Revalidation of the Curiosity and Exploration Inventory-II (CEI-II) using network analysis

**DOI:** 10.3389/fpsyg.2025.1514959

**Published:** 2025-07-30

**Authors:** Sergio Navas-León, Pedro Juan Pérez-Moreno, Carmen Santin Vilarino, Diego Diaz-Milanes

**Affiliations:** ^1^Centro de Investigación Nebrija en Cognición (CINC), Facultad de Lenguas y Educación, Universidad Nebrija, Madrid, Spain; ^2^Department of Clinical and Experimental Psychology, University of Huelva, Huelva, Spain; ^3^Department of Quantitative Methods, Universidad Loyola Andalucía, Sevilla, Spain; ^4^Health Research Institute, University of Canberra, Canberra, ACT, Australia

**Keywords:** curiosity, creativity, CEI-II, psychometric properties, network analysis, Bayesian network, Spain

## Abstract

**Background:**

Curiosity is crucial across various domains, from clinical to educational fields, and holds potential for psychological interventions. Accurate definition and assessment of curiosity are essential for understanding its role and utility. Traditional approaches like factor analysis may not fully capture the construct's nuances.

**Objectives:**

This study aims to reassess the psychometric properties of the Curiosity and Exploration Inventory-II (CEI-II) using Network Analysis.

**Methods:**

A total of 849 Spanish undergraduate students participated in the study. Descriptive analysis, partial-correlation network analysis with gender invariance testing, and Bayesian network model estimation were conducted.

**Results:**

The findings indicate that the CEI-II is best conceptualized as a stable, one-dimensional model, consistent with prior research. The partial-correlation network exhibited moderate density and was invariant in structure, centrality measures, and edge strength across genders, although global strength differed. The Bayesian network identified key pathways for designing interventions based on curiosity.

**Conclusions:**

While the results revealed three distinct item groupings based on centrality measures—challenge-seeking (specific curiosity), novelty-seeking (diversive curiosity), and a combination of both—the empirical evidence supported a stable unidimensional network structure. Items related to specific curiosity showed stronger interconnections, highlighting their importance in fostering curiosity-driven behaviors. These insights suggest that interventions targeting key items may enhance curiosity, and accounting for gender differences could further improve effectiveness.

## 1 Introduction

A century of research highlights that curiosity has been a topic of interest for psychologists, philosophers and educational scientists (Kidd and Hayden, 2015). This has showed that curiosity is vital across various domains of life. In clinical terms, curiosity predicts reduction in anxiety and depression symptoms (Zainal and Newman, 2022), and improved emotional mood (Lydon-Staley et al., 2020). In the workplace, curiosity predict entrepreneurial and workplace innovation (Celik et al., 2016; Peljko et al., 2016; Chang and Shih, 2019) and it is linked to better overall performance (Kashdan et al., 2020). Socially, it is associated with increased positive affect and interpersonal attractiveness (Kashdan and Roberts, 2004). And lastly, in educational contexts, curiosity contributes to higher grades and lower dropout rates (Wavo, 2004; Singh and Manjaly, 2022; Kashdan and Yuen, 2007). Crucially, sociodemographic factors can influence curiosity; for instance, interest in new experiences may declines with age (Mascherek and Zimprich, 2012; Sakaki et al., 2018). Furthemore, regarding sex-at-birth, women typically display higher curiosity about people, while men tend to be more curious about objects (Su et al., 2009; Graziano et al., 2011).

The strong connection between curiosity and these positive outcomes described above underscores the need to explore methods for enhancing curiosity. To address this, various interventions have been developed and refined over time. In this regard, Schutte and Malouff (2023) conducted a comprehensive review of 41 randomized controlled trials involving 4,496 participants, finding that curiosity-enhancing (i.e., mindfulness training or game play) interventions effectively increased curiosity with a moderate effect size (Hedges' *g* = 0.57). In methodological terms, the effectiveness of these interventions is commonly assessed through questionnaires, mainly self-reported instruments designed to measure levels of curiosity. Schutte and Malouff (2023) reported that 80% of studies employed these questionnaires, and Gross et al. (2020) found that nearly 90% of educational interventions aimed at enhancing curiosity also relied on them. Thus, self-report questionnaires are extensively used to assess curiosity levels and are considered essential in the field (Johnson et al., 2023).

In this context, over the last decades several questionnaires have been developed (e.g., Kashdan and Roberts, 2004; Aschieri et al., 2020; Naylor, 1981; Reio et al., 2006) (see Grossnickle, 2016 or Wagstaff et al., 2021, for a review on the topic).

However, this extensive interest in defining the concept and operationalizing it in the form of a questionnaire or scale has resulted in numerous—and often inconsistent—definitions and approaches (Kidd and Hayden, 2015; Grossnickle, 2016). But as recently noted by Jovanović and Gavrilov-Jerković (2014), one of the most widely accepted definitions and explanatory models is the one proposed by Kashdan et al. (2004) and Kashdan et al. (2009) from positive psychology. This model conceptualizes curiosity as a personality trait with two key components: “ Stretching” which refers to the desire to explore and seek out new experiences, and “Embracing” which involves a readiness to accept novel and unfamiliar situations, places, and people (Kashdan et al., 2004, 2009). Given that curiosity drives individuals to seek new experiences and adapt to novelty, it has been described as a positive emotional-motivational system that empowers individuals to thrive and fosters a sense of subjective and psychological wellbeing (Kashdan et al., 2004, 2009).

Following this model, Kashdan et al. (2004) and Kashdan et al. (2009) developed the Curiosity and Exploration Inventory (CEI) which has become one of the most extensively used self-report questionnaires in the field (Balgiu, 2018; Setyowati et al., 2020). However, the original CEI had limitations, notably in capturing the full breadth of the construct and the willingness to manage or embrace the tension associated with novelty and uncertainty (Ye et al., 2015). To address these issues, Kashdan et al. (2009) developed the Curiosity and Exploration Inventory-II (CEI-II), which includes the two subscales: “Stretching,” which measures the motivation to seek knowledge and new experiences, and “Embracing,” which assesses the acceptance of uncertainty and the unpredictable nature of daily life. The refined CEI-II has showed strong reliability and validity across diverse cultural contexts in North America, Europe, Africa, and Asia (e.g., Johnson et al., 2023; Kashdan et al., 2009; Balgiu, 2018; Ye et al., 2015; Tarilonte-Castaño et al., 2023).

Despite its advantages, the CEI-II faces a significant limitation related to its factor structure (Ye et al., 2015). The high correlation between the “Stretching” and “Embracing” subscales raises concerns about their distinctiveness and practical utility. For instance, recent research by Johnson et al. (2023) reported an inter-factor correlation of 1.01^1^, Tarilonte-Castaño et al. (2023) found an inter-factor correlation of 0.97 and Kashdan et al. (2009) noted a high correlation of 0.85. Although Setyowati et al. (2020) reported a lower inter-factor correlation of 0.43, the majority of studies show high values, and often leads to high cross-loadings in exploratory factor analysis (Ye et al., 2015). Given these challenges, some authors report a one-dimensional solution for the CEI-II. In this regard, Tarilonte-Castaño et al. (2023) validated the CEI-II in a sample of Spanish university students, demonstrating that the one-dimensional model exhibits strong psychometric properties and high internal consistency. This conclusion aligns with the traditional latent factor model, which posits that items are influenced by a common underlying construct, such as curiosity, and support causal latent variable theory, which suggests that items are related because they stem from the same latent construct (Costantini et al., 2015).

In addition, Tarilonte-Castaño et al. (2023) found that CEI-II showed a strict invariance between gender even though some studies highlight gender disparities in curiosity. For instance, Su et al. (2009) conducted a meta-analysis showing that men had stronger interests in Realistic (*d* = 0.84) and Investigative (*d* = 0.26) areas, including Engineering (*d* = 1.11) and Science (*d* = 0.36), aligning with the “Stretching” component of the CEI-II, which focuses on seeking new experiences. In contrast, women showed higher interests in Artistic (*d* = −0.35), Social (*d* = −0.68), and Conventional (*d* = −0.33). Women's stronger inclination toward Artistic, Social, and Conventional interests might reflect a tendency to engage with and accept the emotional and social aspects of new experiences, aligning with the “Embracing” component. These findings may suggest that the instrument is suitable for exploring gender-based differences in curiosity; however, it is possible that the sensitivity of the analysis is insufficient to detect subtle violations of invariance that could arise at a more granular level. This could be better understood using alternative methods such as network analysis.

Therefore, an alternative to the traditional factorial model is network analysis, which could offer valuable insights into this issue. Unlike classical approaches, network analysis views psychological phenomena as networks of interconnected characteristics (Borsboom and Cramer, 2013). Recent developments in network psychometrics propose an alternative view of psychological constructs, conceptualizing them as systems of interconnected components rather than reflections of latent variables. From this perspective, traits such as curiosity are understood as emergent properties of direct interactions among observed behaviors or cognitions (Cramer, 2012; Epskamp and Fried, 2018).

This method could help clarify whether “Stretching” and “Embracing” are distinct or overlapping by visualizing item connections, identifying core and peripheral items. It may refine the scale, adjust subscale boundaries, and assess if a unidimensional model is more suitable. Additionally, recent advancements in network analysis could facilitate comparisons of network structures across different populations, offering a deeper understanding of psychological constructs (van Borkulo et al., 2022). This approach could also assess the invariance of items and their interrelationships across genders. Finally, among network-based analyses, we also find Bayesian Networks (BN). These are Directed Acyclic Graphs (DAG) that, when combined with conditional probabilities, can identify pathways among variables to uncover predictors or even causal-effect relationships (Kitson et al., 2023). This analysis could provide a clearer idea of which nodes are key to increasing curiosity in educational or clinical settings, among others, in order to design more effective interventions (Briganti et al., 2022).

The aim of the present study is to re-evaluate the Curiosity and Exploration Inventory-II (CEI-II) using network analysis in a sample of Spanish university students, extending previous work by Tarilonte-Castaño et al. (2023). Prior findings have highlighted challenges in distinguishing the ‘Stretching' and “Embracing” subscales due to high inter-factor correlations, raising concerns about the discriminant validity of the traditional factorial structure. In this context, network analysis offers a valuable alternative to classical latent variable approaches, as it allows for the direct examination of inter-item relationships without assuming underlying common causes.

By estimating both undirected and directed network structures, the study aims to provide a comprehensive representation of the CEI-II's internal organization. This includes evaluating the dimensional structure, the stability of item associations, and the predictability of individual items within the network. Furthermore, the study explores structural invariance across gender, addressing potential item-level differences in network configuration previously suggested in the literature.

Adopting the network psychometric perspective, the CEI-II is conceptualized not as a reflection of latent traits but as a system of interrelated components whose interactions give rise to curiosity-related behaviors. This approach is expected to yield richer insights into the functioning of the scale in this population and to inform the design of future interventions by identifying highly connected or influential items that may serve as leverage points for behavioral change.

## 2 Methods

### 2.1 Recruitment and participants

This study is part of the Health Behavior in University (HBU) project, utilizing a cross-sectional survey approach where participants were recruited using stratified random cluster sampling. For more details of the design and methods conducted in research project, see Andrés-Villas et al. (2020).

The current study included 939 students who consented to participate. After excluding those who did not complete the consent form, minors, age outliers within the university population, and participants/questionnaires with missing values, the final study sample consisted of 849 students. Among them, 74.56% identified as female and 25.44% as male, with an age range of 18–26 years (M = 20.66; SD = 2.151).

### 2.2 Instruments

The *Curiosity and Exploration Inventory (CEI-II)*, created by Kashdan et al. (2009), consists of a 10-item Likert-type scale with five response options ranging from 1 (“Very little or none”) to 5 (“A lot”). A higher score indicates a greater level of curiosity. The Spanish version was developed using a translation and back-translation process conducted by two bilingual translators. This process was supervised by the Spanish team of the WHO Regional Office for Europe's Health Behavior in School-Aged Children Study (Moreno et al., 2020). Semantic equivalence between the original and back-translated versions was ensured through discussion and revision until all discrepancies were resolved (Hambleton and de Jong, 2003). Under traditional factorial analysis approach this adaptation has showed excellent psychometric properties in terms of reliability and validity in the present sample (Tarilonte-Castaño et al., 2023).

### 2.3 Statistical Analysis

All the analyses were performed using R version 4.2.3 (R Core Team, 2018).

#### 2.3.1 Descriptive analysis of the items by gender

First, descriptive analyses were conducted to determine the mean and standard deviation of the CEI-II and its items by gender, as well as their statistically significant differences. Due to the multiple pairwise comparisons, a False Discovery Rate (FDR) correction (Benjamini and Hochberg, 1995) was applied to prevent an increase in Type I error. These analyses were performed using the psych (version 2.2.3), effectsize (version 0.8.9), and dplyr (version 1.1.4) R packages.

#### 2.3.2 Community detection

To evaluate the number, organization, and robustness of the network's dimensional structure, a bootstrap-based Exploratory Graph Analysis (EGA) was performed (Golino et al., 2002). In this approach, variable communities—representing distinct dimensions—are identified by detecting clusters of nodes that exhibit strong mutual associations through connecting edges. These clusters reflect coherent groups of variables that tend to function together. Items were assigned to a given community only if their stability coefficients exceeded 0.70, ensuring reliability in their classification. Items with lower stability were excluded to prevent potential disruption of the dimension's structural integrity (Christensen and Golino, 2021). The R package used for this analysis was EGAnet (version 2.0.2).

#### 2.3.3 Network structure estimation

To estimate the structural configuration of the CEI-II item network, a partial-correlation approach was employed within the framework of Gaussian Graphical Models (GGMs) (Epskamp and Fried, 2018; Epskamp et al., 2018). In this model, edges between nodes represent conditional dependencies that persist after controlling for the influence of all other variables. To enhance interpretability and reduce spurious connections, the network was regularized using the Least Absolute Shrinkage and Selection Operator (LASSO), which systematically eliminates weak associations by shrinking them to zero (Friedman et al., 2008; Van Borkulo et al., 2014). This yields a more parsimonious and robust representation of the network. The reliability of the estimated edge weights was evaluated through the computation of confidence intervals, typically at the 95% level (Epskamp and Fried, 2018; Hevey, 2018). All analyses were conducted using the R packages qgraph (version 1.9.5), for network estimation, and mgm (version 1.2.14), to characterize its predictability power.

#### 2.3.4 Estimation of centrality measures

Centrality indices quantify the importance of nodes within a network, reflecting their relative influence in the model. In this study, four centrality measures were computed: strength, closeness, betweenness, and expected influence (Epskamp and Fried, 2018; Epskamp et al., 2018; Opsahl et al., 2010). Strength represents the sum of edge weights connected to a node, closeness captures the inverse of the total distance from one node to all others, and betweenness measures how frequently a node lies on the shortest paths between other nodes (Epskamp et al., 2018; Opsahl et al., 2010).

To evaluate the reliability of these indices, a Correlation Stability (CS) coefficient was calculated using subset bootstrapping with 1,000 samples. This metric indicates the maximum proportion of observations which can be removed while still maintaining a correlation of 0.70 or higher between the original centrality indices and those of the subsets, with 95% certainty (Epskamp and Fried, 2018). A CS coefficient above 0.25 was considered acceptable, with values above 0.50 indicating good stability (Hevey, 2018; Glück et al., 2017). All analyses were conducted in R using the packages qgraph (version 1.9.5), for centrality estimation, and bootnet (version 1.5.6), for assessing the stability of centrality metrics.

#### 2.3.5 Analysis of invariance in network structure

To assess whether the network structure was invariant across gender groups, a Network Comparison Test (NCT) was conducted (van Borkulo et al., 2022). It is important to note that this test assesses differences in structural associations (i.e., partial correlations between items), not measurement relations as defined in latent variable models. Therefore, the results are interpreted in terms of structural rather than measurement invariance. This procedure evaluates potential differences between groups at four distinct levels: (1) overall network structure, based on the assumption that both networks share the same configuration; (2) global strength, referring to the total level of connectivity across the network; (3) individual edge strength, which examines whether the magnitude of specific connections between nodes differs between male and female networks; and (4) centrality measures, which were compared based on their Correlation Stability (CS) coefficients. The Benjamini-Hochberg procedure was applied to correct *p*-values for multiple testing in the analysis of edge differences. The R package used for this analysis was NetworkComparisonTest (version 2.2.2).

#### 2.3.6 Bayesian network estimation

The development of the Bayesian Network (BN) followed a two-step procedure. The first step involved estimating the underlying Directed Acyclic Graph (DAG), and the second focused on fitting and validating the BN model using the study dataset. DAG estimation was performed using the PC-Stable algorithm without applying any constraints (Scutari, 2010). The algorithm starts with a complete graph and removes edges based on conditional independence tests. V-structures are identified, and directional edges are added if no cycles exist, based on correlation data, which limits causal conclusions (Li et al., 2019; Spirtes and Glymour, 1991). To enhance the robustness of the resulting structure, 200 bootstrap samples were generated, and only edges with a selection frequency above 0.85 and a directionality >0.5 were retained in the final graph (Briganti et al., 2022).

In the second step, the dataset was subjected to a 10-fold cross-validation procedure to fit and validate the BN model. In each iteration, 90% of the data was used for model training and the remaining 10% for testing, repeating this process until all fold combinations had been evaluated.

The implementation of this procedure was carried out using the bnlearn R package (version 4.9) for DAG estimation, dismo (version 1.3-14) for generating the k-fold partitions, and caret (version 6.0.94) for evaluating the predictive performance of the model.

#### 2.3.7 Predictive performance

Model performance in terms of predictive accuracy and error was evaluated using two metrics: the coefficient of determination (R^2^) and the root-mean-square error (RMSE). These measures were applied to both the partial-correlation networks and the Bayesian Network (BN) model, providing insights into both absolute and relative accuracy. In the partial-correlation framework, a node's predictability was estimated by treating all other nodes in the network as potential predictors (Haslbeck and Fried, 2017). In contrast, within the BN approach, the predictability of a target node (child) was assessed based solely on its direct parent nodes, as defined by the directional connections identified during DAG construction.

Predictive accuracy and error for each node were calculated as the average values across all iterations of the cross-validation procedure implemented in the second phase of the BN modeling. In both modeling approaches, predictability was visually represented in the network diagrams using node borders, with variations in appearance indicating different levels of explained variance. Performance metrics were obtained using the mgm package (version 1.2.14) for the GGM and the caret package (version 6.0.94) for the BN model.

For additional detail on the use of these metrics in network analysis and Bayesian networks, see Scutari (2010), Borsboom et al. (2021), and Haslbeck et al. (2021).

## 3 Results

### 3.1 Descriptive statistics

Table 1 presents the means and standard deviations by gender for the 10 items and the overall scale score. Statistically significant differences (*p* < 0.05) were identified in every item except for items 1 and 10. Females scored higher on items 2 and 8 but lower on the rest. Regarding the total score, significant differences were found, with males scoring higher than females, although the effect size was small.

**Table 1:** Descriptive statistics of the CEI-II items by gender.

**Item**	**Male M (*SD*)**	**Female M (*SD*)**	**Student's *T***	***p*-value (bilateral)**	**Effect size**
1. I actively seek as much information as I can in new situations.	3.639 (0.904)	3.518 (0.902)	1.695 (371.147)	0.091	0.134
2. I am the type of person who really enjoys the uncertainty of everyday life.	3.259 (1.086)	2.75 (1.125)	5.892 (383.989)	< 0.001	0.457
3. I am at my best when doing something that is complex or challenging.	3.806 (0.969)	3.444 (1.011)	4.684 (386.373)	< 0.001	0.362
4. Everywhere I go, I am out looking for new things or experiences.	3.736 (0.91)	3.561 (0.981)	2.396 (398.03)	0.017	0.182
5. I view challenging situations as an opportunity to grow and learn.	4.023 (0.849)	3.761 (0.901)	3.851 (392.373)	< 0.001	0.295
6. I like to do things that are a little frightening.	3.356 (1.124)	3.133 (1.126)	2.526 (372.556)	0.012	0.199
7. I am always looking for experiences that challenge how I think about myself and the world.	3.56 (0.991)	3.245 (1.102)	3.921 (409.711)	< 0.001	0.294
8. I prefer jobs that are excitingly unpredictable.	3.338 (1.044)	2.954 (1.069)	4.637 (379.698)	< 0.001	0.362
9. I frequently seek out opportunities to challenge myself and grow as a person.	3.773 (0.969)	3.494 (1.019)	3.601 (389.022)	< 0.001	0.277
10. I am the kind of person who embraces unfamiliar people, events, and places.	3.028 (1.169)	3.041 (1.271)	−0.141 (401.207)	0.888	0.011
Total score	35.519 (7.12)	32.902 (7.3)	4.634 (380.286)	< 0.001	0.361

*M*, mean; *SD*, standard deviation; effect size is calculated as Cohen's d, where values of 0.2, 0.5, and 0.8 are considered small, medium, and large, respectively. A *p* < 0.05 is considered statistically significant.

### 3.2 Network analysis

#### 3.2.1 Bootstrap exploratory graph analysis

**Figure 1:**
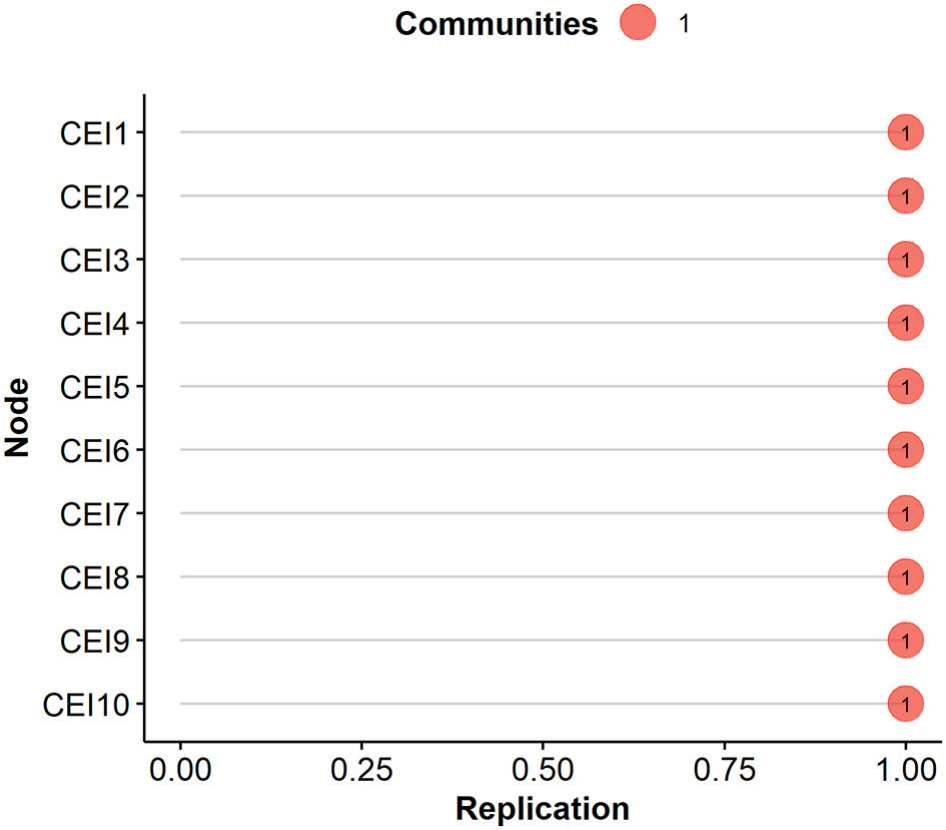
Community stability coefficients for CEI-II items.

The bootstrap exploratory graph analysis identified a one-community solution with 100% probability. Furthermore, all items loaded onto this single community with 100% stability, as supported by the 95% confidence intervals of the edges—indicating both strong interpretability and high accuracy of the network structure (Figure 1). Regarding the centrality measures, the strength, closeness, and betweenness of the obtained network were found to be stable, with CS-coefficients of 0.75 (min = 0.673; max = 1), 0.595 (min = 0.517; max = 0.673), and 0.439 (min = 0.362; max = 0.517), respectively. Therefore, the network structure can be considered unidimensional, and its edges and centrality indices are fully reliable.

**Figure 2:**
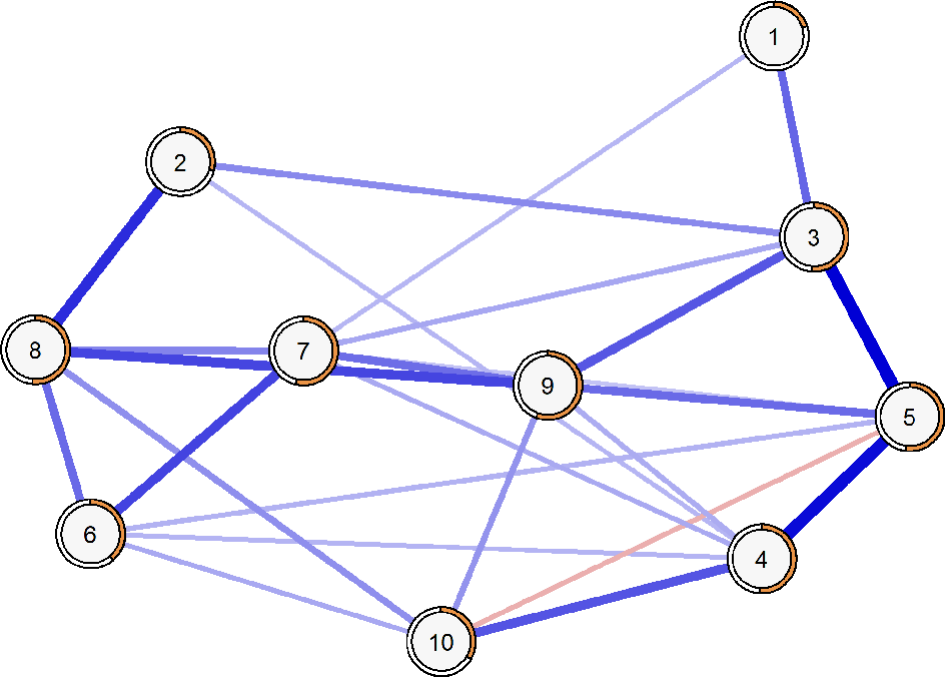
Partial-correlation network showing relationships between items of CEI-II. R2 is represented with orange borders.

Figure 2 displays the partial-correlation network structure of the CEI-II, where almost all identified relationships are represented in blue due to positive correlations among the nodes, except for the edge between nodes 5 and 10, represented in red due to their inverse relationship. The network density was 55.56% (25 non-zero edges out of 45 possible edges) and showed an average weight of 0.164, with a minimum of −0.11 and a maximum of 0.34. The most robust connections were identified between item 5 and items 3 and 4, both with weights >0.3, while the least robust connection, in absolute values, was between item 5 and item 7, with a value lower than 0.1.

**Figure 3:**
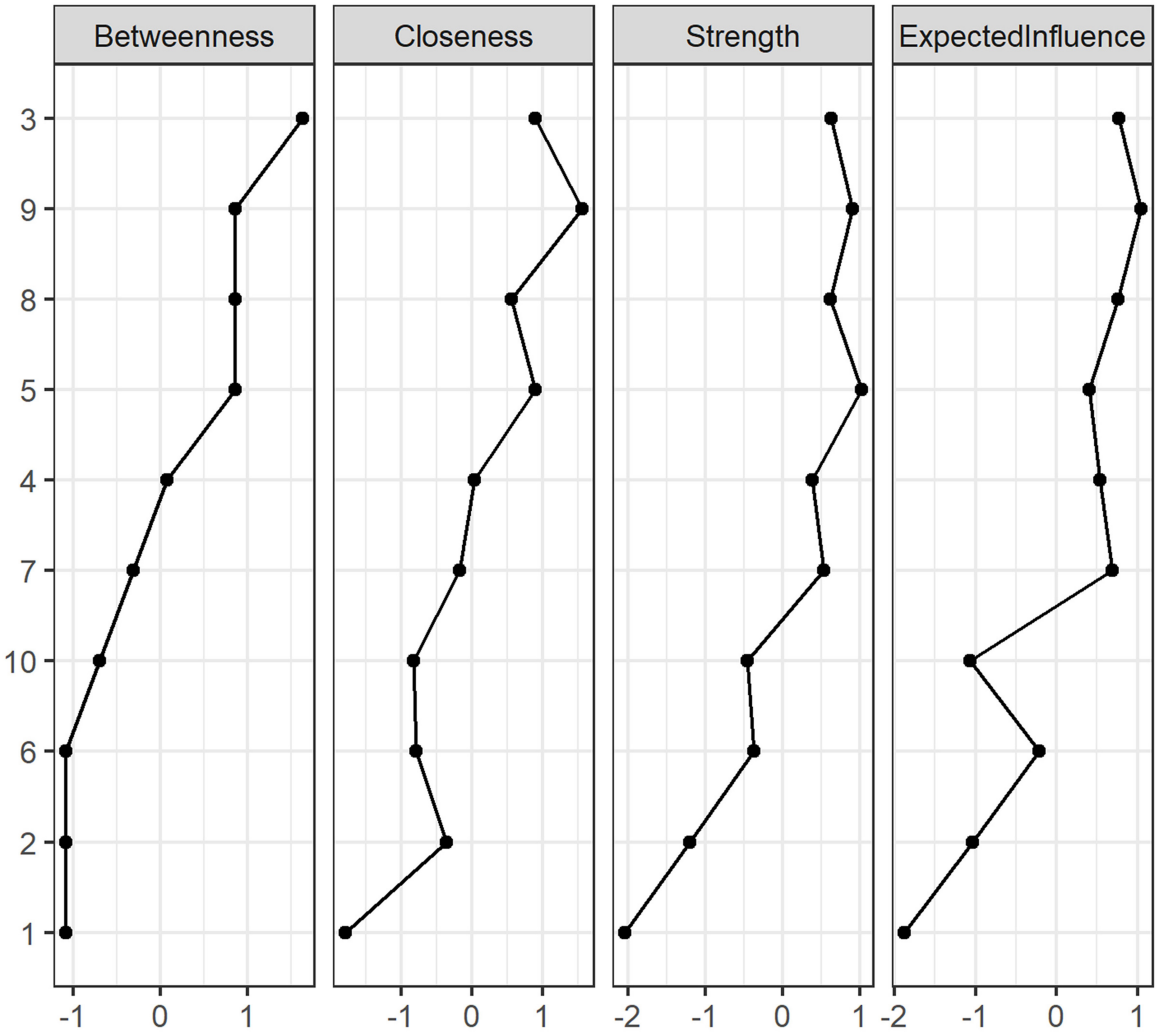
Standardized centrality measures of the network (betweenness, closeness, strength and expected influence).

Regarding centrality measures (Figure 3), item 5 exhibits the highest strength value, closely followed by item 9. In terms of betweenness, item 3 has the highest value, followed by items 5, 8, and 9, which hold most of the indirect paths among nodes in the network. For closeness, the highest score was obtained by item 9, followed by items 3 and 5. Across all indicators, item 1 had the lowest score, making it the least central node in the network. Expected influence scores were very similar to strength due to the low number of negative connections identified among nodes.

#### 3.2.2 Network structure invariance between males and females

**Figure 4:**
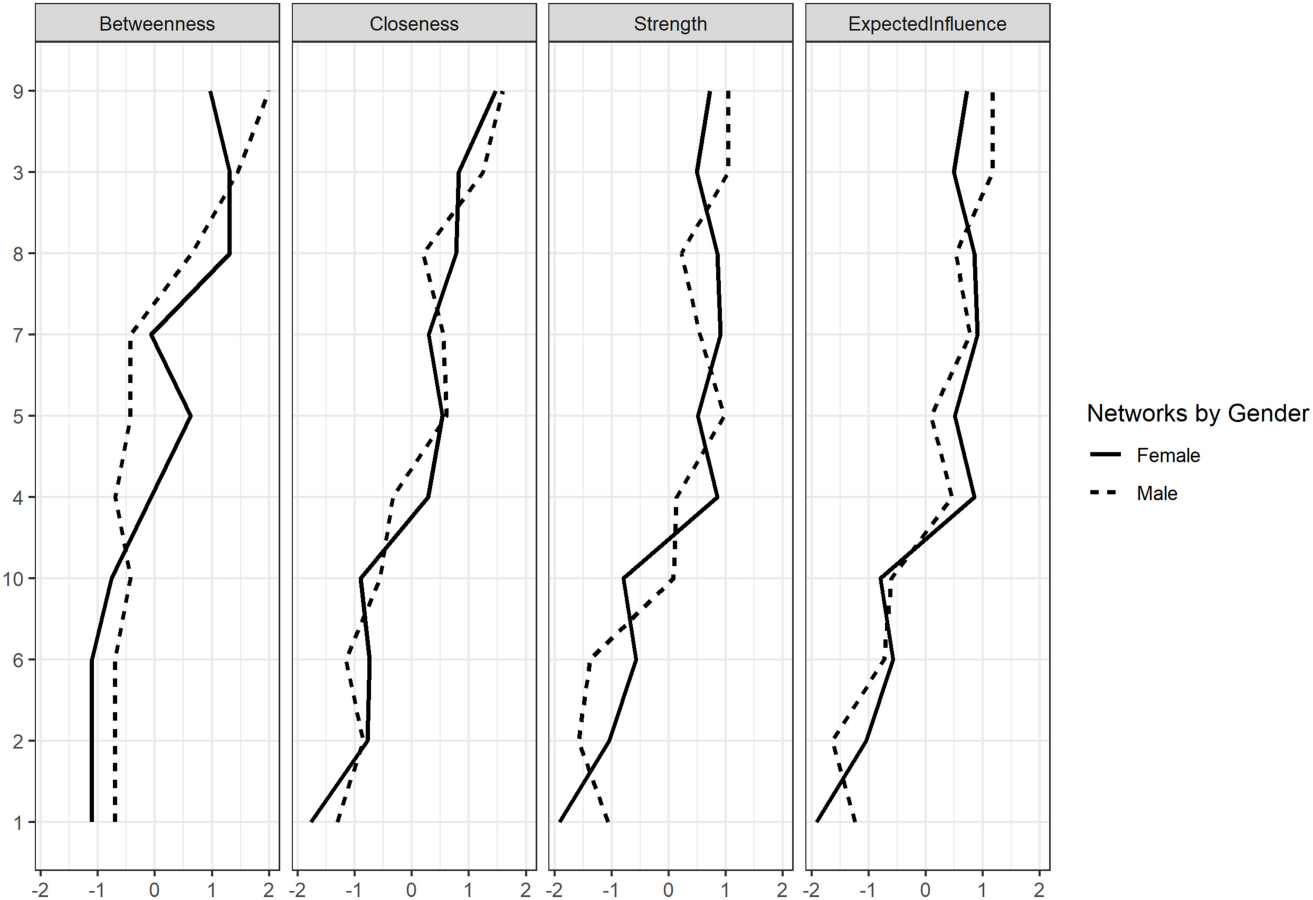
Standardized centrality indices of females and males networks.

**Figure 5:**
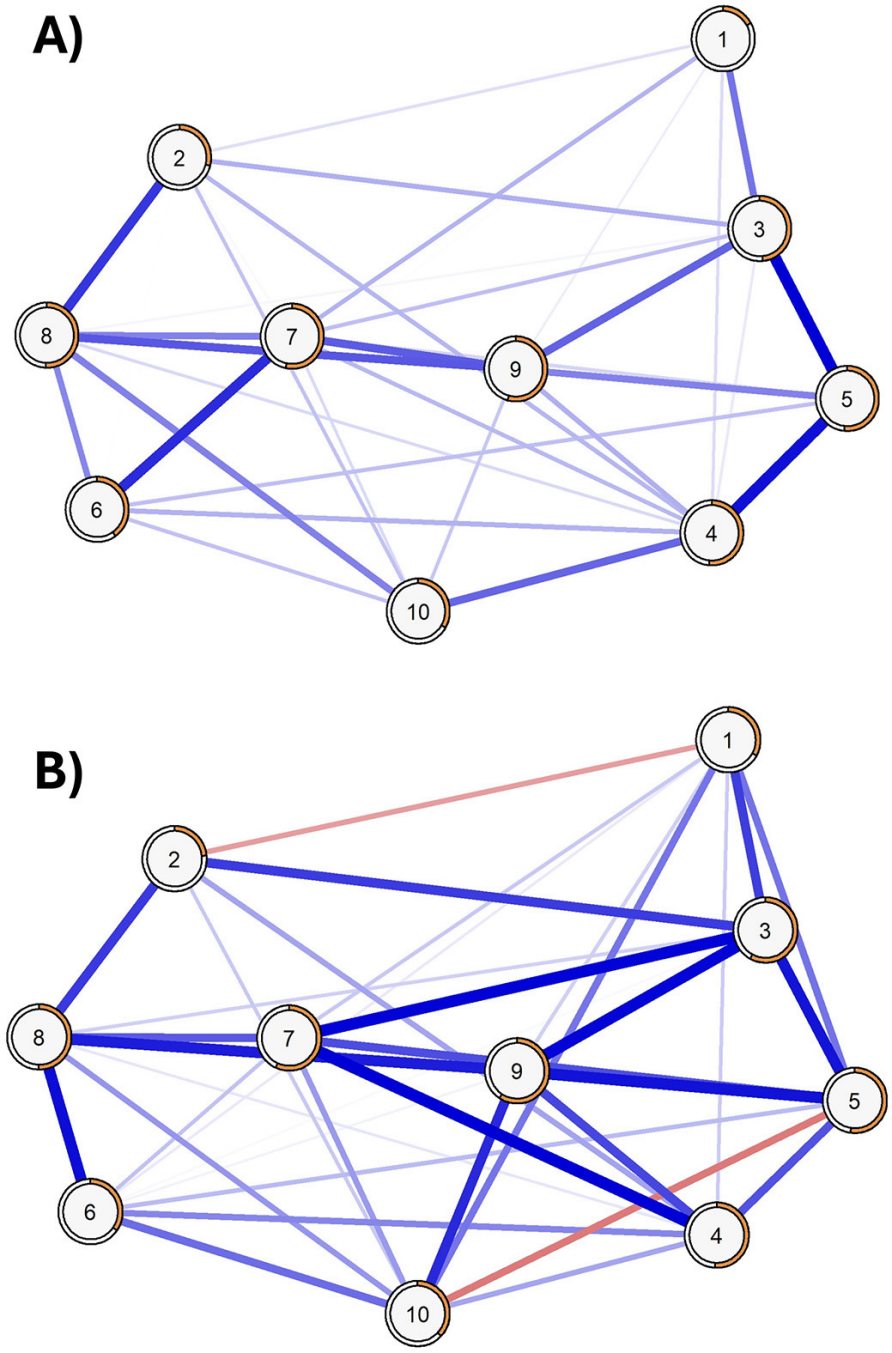
Networks of female [**(A)** panel; *N* = 633] and male participants [**(B)** panel; *N* = 216]. R2 is represented with orange borders.

The Network Comparison Test (NCT) revealed that the female network exhibited lower global strength (global strength = 4.157) than the male network (global strength = 4.57), with the difference reaching statistical significance (S = 0.412, p = 0.045), indicating higher overall connectivity in the male network. In contrast, the difference in network structure (M = 0.203, *p* = 0.21) was not statistically significant, suggesting comparable structural configurations across gender. Additionally, no significant differences were observed in centrality measures (*p* > 0.5 for all comparisons, Figure 4) or edge strength (Figure 5) after applying the Benjamini-Hochberg correction, with a significance threshold set at 0.05.

### 3.3 Bayesian network

#### 3.3.1 DAG estimation

**Figure 6:**
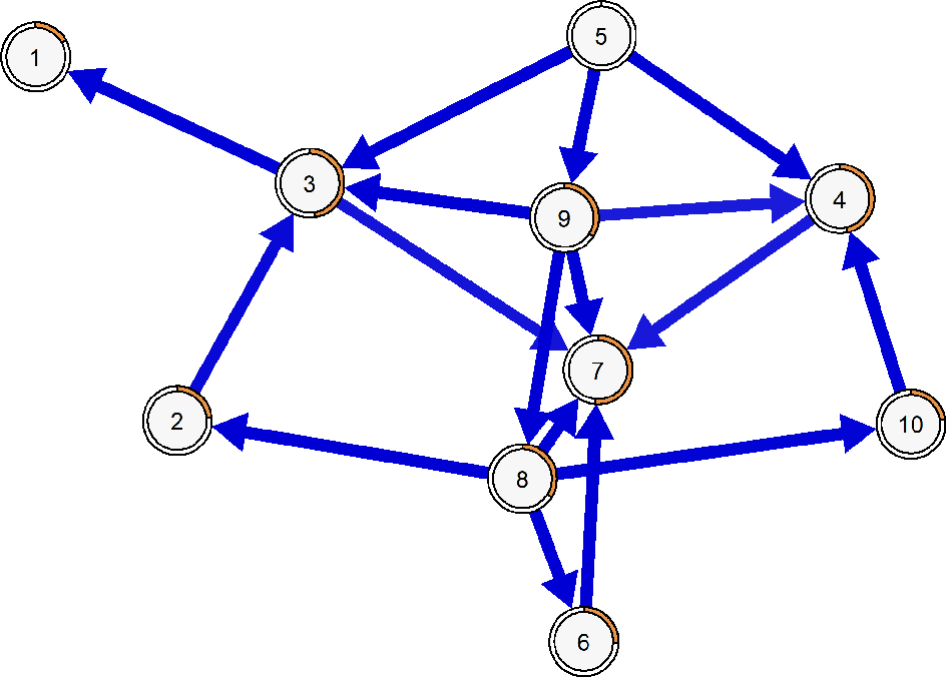
Estimated Bayesian Network for CEI-II items. R2 is represented with orange borders.

The BN was used to determine the likely direction of edges within the network. The final BN consisted of a DAG containing 16 directed edges. In this structure, item 5 served as the sole parent node, initiating the network. In contrast, items 1 and 7 functioned exclusively as child nodes, while the remaining items played a dual role, acting both as parent and child nodes within the network (see Figure 6).

#### 3.3.2 Models predictability

The predictability and error for each item in the network were estimated using partial-correlation models for the total of the sample, females and males separately (see Table 2). In every model, item 9 was the one with the highest explained variance and lower RMSE, while item 1 showed the lowest explained variance and highest RMSE. Furthermore, item 1 showed the greatest discrepancy by gender, with a 0.16 in *R*^2^ among them, being the female group the one with worse predictability.

**Table 2:** Explained variance and RMSE of partial-correlation network (GGM) and BN models.

**Variable**	**GGMs**						**BN**	
	**General**		**Female**		**Male**			
	*R* ^2^	**RMSE**	*R* ^2^	**RMSE**	*R* ^2^	**RMSE**	*R* ^2^	**RMSE**
Item 1	0.201	0.893	0.17	0.91	0.33	0.816	0.169	0.826
Item 2	0.291	0.842	0.292	0.841	0.234	0.873	0.237	0.993
Item 3	0.515	0.696	0.486	0.716	0.581	0.646	0.477	0.731
Item 4	0.511	0.699	0.515	0.696	0.509	0.699	0.467	0.703
Item 5	0.52	0.692	0.516	0.695	0.523	0.689	–	–
Item 6	0.387	0.783	0.402	0.773	0.347	0.806	0.256	0.975
Item 7	0.538	0.68	0.535	0.681	0.57	0.654	0.515	0.749
Item 8	0.516	0.695	0.507	0.702	0.505	0.702	0.34	0.872
Item 9	0.558	0.664	0.544	0.675	0.594	0.636	0.341	0.823
Item 10	0.335	0.815	0.335	0.815	0.37	0.792	0.227	1.101

Regarding the predictability of the BN model, it showed the highest explained variance for item 7, which receives the most influence from the rest of the network, while the lowest error was for item 4. On the other hand, item 1 exhibits the worst predictability, while item 10 showed the largest error in prediction (see Table 2).

## 4 Discussion

The aim of the present study was to apply psychometric network analysis to investigate the network model of the Spanish version of the CEI-II, including potential differences between females and males, and to analyze the explanatory power and accuracy of the models. To the best of our knowledge, this study is the first to apply network analysis to the CEI-II.

### 4.1 Factor structure and network perspective: complementary approaches

Regarding the factor and network structure, from a quantitative standpoint, the results are best understood as supporting a stable unidimensional model. This finding aligns with the factor structure identified in the analysis conducted by Tarilonte-Castaño et al. (2023), providing converging evidence for the model. This model is a more parsimonious solution than the one obtained in the original study (Kashdan et al., 2009), demonstrating that these communities are consistent with the latent factors of factor models (Golino and Epskamp, 2017).

This unidimensionality raises questions about the suitability of applying network analysis vs. traditional factorial models. While factor analysis assumes that items reflect an underlying latent variable, network analysis conceptualizes items as dynamic entities that interact with each other. In this sense, the observed unidimensional structure could indicate that the CEI-II items are strongly interconnected and form a cohesive system, justifying the application of network analysis to examine the relationships between items beyond a traditional factorial structure. This finding suggests that curiosity, as measured by the CEI-II, can be examined both as a latent construct and as a system of interacting items, depending on the analytical framework. The network approach emphasizes item-level dynamics and potential intervention targets in relation to theoretical models, supporting their construction and redefinition. As such, these models can be viewed as a complementary analytical perspective to the factor analysis reported by Tarilonte-Castaño et al. (2023), enabling different levels of granularity and facilitating more precise and effective interventions or evaluations.

### 4.2 Item-level patterns

At the item level, in terms of betweenness (i.e., the extent to which an item lies on the shortest paths between other items), closeness (i.e., the measure of how quickly an item can access other items in the network), and strength (i.e., the sum of the weights of the items connected to an item), three groups of items can be visually identified, attending to the aforementioned metrics. However, it should be noted that this interpretative perspective, at item level, was established *post-hoc* and does not reflect distinct empirical dimensions. The robust unidimensional solution remains, with the groupings offering a conceptual guide for more targeted interventions based on item content and centrality measures.

First, there are items related with challenge-seeking such as Item 5 (“*I view challenging situations as an opportunity to grow and learn*”), Item 9 (“*I frequently seek out opportunities to challenge myself and grow as a person*”), Item 3 (“*I am at my best when doing something that is complex or challenging*”) and Item 8 (“*I prefer jobs that are excitingly unpredictable*”). This group of items may align with the “Embracing” dimension of the CEI-II, as outlined by Kashdan et al. (2009).

From an epistemic view, the aforementioned items may comprise the so-called “specific curiosity” (Berlyne, 1960). Specific curiosity can be defined as exploration that leads to a detailed investigation of new stimuli to obtain new information (Schutte and Malouff, 2023; Berlyne, 1960; Litman and Spielberger, 2003). This type of curiosity, built on complexity theories (see Dubey and Griffiths, 2020 for a review), is deliberate, goal-oriented and closely linked to problem-solving, helping to resolve knowledge gaps by acquiring substantive facts (Grossnickle, 2016; Hagtvedt et al., 2019; Harrison et al., 2011; Le Cunff, 2024). Thus, individuals with high specific curiosity are prone to seek out situations and events that are notably ambiguous or uncertain (Kashdan et al., 2009). Ultimately, it has been demonstrated that this process can foster creativity (Hagtvedt et al., 2019).

Second, there are items related with novelty-seeking dimension of curiosity such as Item 7 (“*I am always looking for experiences that challenge how I think about myself and the world*”), Item 4 (“*Everywhere I go, I am out looking for new things or experiences*”), and Item 10 (“*I am the kind of person who embraces unfamiliar people, events, and places*”). This group of items may align with the “Stretching” dimension of CEI-II, as outlined by Kashdan et al. (2009).

The aforementioned items may comprise the so-called “diversive curiosity” (Berlyne, 1960). Diversive curiosity is commonly described as the tendency to seek stimulation regardless of source or content (Berlyne, 1960; Litman and Spielberger, 2003). This type of curiosity, build on novelty theories (see Dubey and Griffiths, 2020 for a review), it is spontaneous, driven by the desire of boredom, novelty or stimulation (Schutte and Malouff, 2023; Kashdan et al., 2009; Litman and Spielberger, 2003; Le Cunff, 2024; Whitecross and Smithson, 2023). Individuals with high diversive curiosity actively are ready to grow and expand, rather than remaining in familiar situation (Chang and Shih, 2019). This type of curiosity can lead to high distractibility, with individuals frequently switching between topics and activities due to sudden interest in something new and unrelated (Whitecross and Smithson, 2023); and ultimately can lead to unlearned situations (Dubey and Griffiths, 2020).

Third, we find items that combine specific curiosity and diversive curiosity such as Item 6 (“*I like to do things that are a little frightening*”) Item 2 (“*I am the type of person who really enjoys the uncertainty of everyday life*”) and Item 1 (“*I actively seek as much information as I can in new situations*”).

Crucially, their weaker centrality measures—such as lower betweenness, closeness and strength—can be interpreted as peripheral items in the network (Hevey, 2018), rather than being key drivers of curiosity. It should be noted for the reader that Item 1 (“I actively seek as much information as I can in new situations”) is the most disconnected in the network. These groupings should be understood as descriptive patterns based on item centrality within a unified curiosity network, not as distinct latent dimensions (Borsboom and Cramer, 2013; Robinaugh et al., 2016). This perspective is compatible with the unidimensional structure identified through EGA (Christensen and Golino, 2021; Golino and Epskamp, 2017). Thus, these groupings are not psychometrically validated subtypes, but rather a conceptual framework that may guide future research and interventions (Fried and Cramer, 2017).

### 4.3 Gender differences in network structure

In the analysis from a gender perspective, item-level results from the Network Comparison Test (NCT) revealed no significant differences in the overall network structure, centrality measures, or edge strength between males and females. However, a significant difference in global strength was found, with the male network showing greater overall connectivity than the female network. Differences in network structure or global strength between gender groups are interpreted as variations in the organization of item-level associations, consistent with the network psychometric perspective. These findings do not imply violations of measurement invariance in the classical psychometric sense but rather suggest potentially distinct patterns of inter-item activation between groups.

One possible explanation is that males are generally less risk-averse and more tolerant of uncertainty in decision-making, which may result in more feedback loops and interconnected nodes within their curiosity network (Robichaud et al., 2003; Harris and Jenkins, 2006). However, it is important to note that uncertainty is context-dependent, and various situational factors can influence how risk aversion manifests across genders (Schubert et al., 1999). Additionally, research suggests that males tend to be more motivated by goal-directed tasks and exhibit greater exploratory behavior in novel situations compared to females (Sarin and Wieland, 2016). In a recent study, 73.7% of males chose more challenging tasks when given limited options, compared to only 27.5% of females, reflecting higher engagement in challenge-seeking behavior (Bieberstein et al., 2020).

As suggested by Borsboom and Cramer (2013), network analysis allows for interpreting observed variables (e.g., items) not merely as expressions of an underlying latent factor but as components within a causal system characterized by continuous mutual influence. This approach has significant implications for psychological interventions, indicating that targeting central items can enhance therapeutic outcomes.

### 4.4 Bayesian network, item predictability, and error analysis

In this regard the Bayesian Network (BN) analysis reveals important directional insights. While these structural patterns do not imply causality, the results revealed that viewing challenges as opportunities for growth (Item 5) may function as a “parent” node and occupies a central position within the network structure. Item 1 and Item 7, which involve actively seeking information and new stimulus are categorized as “child” nodes and may play a more peripheral role within the network. The remaining items illustrate a network where various items mutually reinforce one with another.

Given the above, items associated with specific curiosity demonstrate stronger connections and may act as key drivers. In contrast, the remaining items appear more peripheral, indicating that not all dimensions of curiosity are equally integrated into the CEI-II. These items might be particularly useful in interventions aimed at improving curiosity. This needs to be empirically tested, however, as we did not conduct formal redundancy diagnostics. Thus, any considerations about item refinement remain preliminary and should be addressed in future studies using dedicated methods. Interestingly, Schutte and Malouff (Schutte and Malouff, 2023) found in their meta-analysis that interventions targeting general curiosity (*n* = 2) showed larger effect sizes compared to those focused on specific curiosity (*n* = 39). Since more interventions focus on specific curiosity, these findings could provide valuable insights for a broad range of studies.

Furthermore, the analysis of predictability and error indicates that Item 9 emerges as the most predictable item, showcasing the highest explained variance and the lowest error. This indicates that it is a central component of the CEI-II. In contrast, Item 1 shows the lowest explained variance and the highest error, particularly within the female sample, suggesting its predictability is influenced by gender differences in information-seeking behavior. Item 7, a “child” node, exhibits the highest explained variance in the BN model, possibly due to receiving the most connections. Lastly, Item 10, shows the largest prediction error, suggesting it may be affected by external factors not adequately captured in the CEI-II network structure.

### 4.5 Limitations and future directions

Nevertheless, there are several limitations that should be recognized. First, our analyses suggest that the network structure is invariant across genders, supporting the generalizability of the overall pattern of relationships. Differences in edge strengths or prevalence estimates could exist when compared with other university student populations, non-university sectors, or in cultures with different gender distributions. Therefore, the gender imbalance could influence certain aspects of the results. Second, the cross-sectional design of the study prevents us from establishing causal relationships among the items, underscoring the need for longitudinal studies and experimental designs to test the obtained models. Third, the network structure occasionally displays a lack of stability, which may lead to inconsistent findings. Fourth, focusing exclusively on the CEI-II may limit our understanding of curiosity as a multidimensional construct; examining other related (and unrelated) constructs could yield additional evidence of validity. Further studies should consider including motivation types and behaviors related to them (e.g., leisure activities) to integrate this construct into a more complex system that is closer to reality. And lastly, we did not conduct formal redundancy diagnostics, so any considerations regarding item overlap or refinement remain preliminary. Future research should address this using dedicated methods (Christensen et al., 2023).

## 5 Conclusion

The findings indicate that the CEI-II is best conceptualized as a stable, one-dimensional model, which aligns with previous research. From an interpretative perspective, the results reveal three distinct groups of items regarding their centrality measures: challenge-seeking (specific curiosity), novelty-seeking (diversive curiosity), and a combination of both. Items related with specific curiosity show stronger interconnections within the network, suggesting they play a pivotal role in fostering curiosity-driven behaviors. This suggests that targeting these key items in interventions may significantly enhance individuals' curiosity. In this regard, to design more effective interventions aimed at fostering curiosity, it might be useful to consider the relevance of different items. Specifically, interventions may prioritize Items 5 and 9, which are identified as central to the network, while Items 1 and 10 appear comparatively less relevant. Additionally, it is essential to address the differences in responses between males and females for more tailored interventions.

## Data Availability

The raw data supporting the conclusions of this article will be made available by the authors, without undue reservation.
